# A serralysin-like protein of *Candidatus* Liberibacter asiaticus modulates components of the bacterial extracellular matrix

**DOI:** 10.3389/fmicb.2022.1006962

**Published:** 2022-10-19

**Authors:** Lucila Garcia, Maria Celeste Molina, Kaylie Allyson Padgett-Pagliai, Pablo S. Torres, Roberto E. Bruna, Eleonora García Véscovi, Claudio F. González, Jose Gadea, María Rosa Marano

**Affiliations:** ^1^Instituto de Biología Molecular y Celular de Rosario (IBR), Consejo Nacional de Investigaciones Científicas y Tecnológicas (CONICET), Rosario, Argentina; ^2^Área Virología, Facultad de Ciencias Bioquímicas y Farmacéuticas, Universidad Nacional de Rosario (UNR), Rosario, Argentina; ^3^Department of Microbiology and Cell Science, Genetics Institute, Institute of Food and Agricultural Sciences, University of Florida, Gainesville, FL, United States; ^4^Instituto de Biología Molecular y Celular de Plantas (IBMCP), Universidad Politécnica de Valencia-C.S.I.C, Ingeniero Fausto Elio, Valencia, Spain

**Keywords:** biofilm, Huanglongbing, protease, surrogate bacteria, virulence factor

## Abstract

Huanglongbing (HLB), the current major threat for *Citrus* species, is caused by intracellular alphaproteobacteria of the genus *Candidatus* Liberibacter (*Ca*L), with *Ca*L asiaticus (*C*Las) being the most prevalent species. This bacterium inhabits phloem cells and is transmitted by the psyllid *Diaphorina citri*. A gene encoding a putative serralysin-like metalloprotease (CLIBASIA_01345) was identified in the *C*Las genome. The expression levels of this gene were found to be higher in citrus leaves than in psyllids, suggesting a function for this protease in adaptation to the plant environment. Here, we study the putative role of *C*Las-serralysin (Las1345) as virulence factor. We first assayed whether Las1345 could be secreted by two different surrogate bacteria, *Rhizobium leguminosarum* bv. *viciae* A34 (*A34*) and *Serratia marcescens*. The protein was detected only in the cellular fraction of *A34* and *S. marcescens* expressing Las1345, and increased protease activity of those bacteria by 2.55 and 4.25-fold, respectively. In contrast, Las1345 expressed in *Nicotiana benthamiana* leaves did not show protease activity nor alterations in the cell membrane, suggesting that Las1345 do not function as a protease in the plant cell. Las1345 expression negatively regulated cell motility, exopolysaccharide production, and biofilm formation in *Xanthomonas campestris* pv. *campestris* (*Xcc*). This bacterial phenotype was correlated with reduced growth and survival on leaf surfaces as well as reduced disease symptoms in *N. benthamiana* and *Arabidopsis.* These results support a model where Las1345 could modify extracellular components to adapt bacterial shape and appendages to the phloem environment, thus contributing to virulence.

## Introduction

Huanglongbing (HLB), also known as citrus greening disease, is a global threat to citrus production (Bové, 2006; Gottwald, 2010; Da Graça et al., 2016). The disease is caused by phloem-limited intracellular Gram-negative alphaproteobacteria belonging to the order of Rhizobiales, family *Rhizobiaceae*, and genus *Candidatus* Liberibacter (*Ca*L; Duan et al., 2009; Kuykendall et al., 2012a; Wang et al., 2017). Three *Ca*L species have been found to associated with HLB, *Ca*L asiaticus (*C*Las), *Ca*L africanus (*C*Laf) and *Ca*L americanus (*C*Lam; Bové, 2006). Currently, *C*Las is the most widespread and virulent HLB-associated pathogen worldwide (Bassanezi et al., 2020; Li et al., 2020). *C*Las is transmitted in a circulative-propagative manner by its insect vector, the Asian citrus psyllid (ACP) *Diaphorina citri* Kuwayama (Hemiptera: Liviidae) during feeding on the phloem of the new shoots (Hall et al., 2016; Lopes and Cifuentes-Arenas, 2021). Upon phloem injection, *C*Las is confined to sieve elements until the young flush turns into mature leaves (source tissues), then it moves intercellularly through sieve pores following phloem sap from source to sink, including roots and new shoots (Andrade and Wang, 2019; Alves et al., 2021; Pandey et al., 2021; Raiol-Junior et al., 2021). Symptoms of *C*Las-infected citrus leaves are associated with an inefficient defense response that affect the structure and function of the phloem sieve elements and would normally confine the invading pathogen (Koh et al., 2012; Boava et al., 2017; Granato et al., 2019). However, callus deposition and starch accumulation were not observed in roots despite high bacterial accumulation in this tissue (Etxeberria et al., 2009; Johnson et al., 2014; Pulici et al., 2022). Moreover, the expression levels of both *CALLOSE SYNTHASE 7* (*CalS7*) and *PLHOEM LECTIN* (*PP2*) were significantly downregulated in HLB-infected roots compared to healthy roots (Achor et al., 2020). Those results support recent investigations which postulate the root system as the primary replication place of *C*Las at the early infection stages (Alves et al., 2021; Pulici et al., 2022). This differential response between leaves and root may reflect a fine control of plant responses by the pathogen, keeping the balance between defense and nutrient-acquisition (Wang et al., 2016; Martinelli and Dandekar, 2017; Chen et al., 2022).

Bacteria are well known for using flagella to swim through liquids, to swarm across solid surfaces, or to promote host colonization through adherence and biofilm formation (Malamud et al., 2011; Chaban et al., 2015; Rossi et al., 2018). Little is known about systemic movement of *Ca*L spp. inside the phloem. Ultra-microscopic analysis of *C*Las-infected plants showed that unflagellated spherical bacterium cells float freely in the phloem sap by changing morphology from spherical to elongated shape in order to cross the sieve pores without attaching to the sieve tube cell walls or to each other, all without forming biofilms (Achor et al., 2020). However, the presence of *Ca*L-associated flagella-like surface structures and aggregates of long rod-shaped cells has been noted inside its psyllid vectors (Cicero et al., 2016; Andrade et al., 2020). Flagella are a highly conserved microbe-associated molecular patterns (MAMPs/PAMPs), so unflagellated *C*Las could potentially avoid the elicitation of a strong PAMP-triggered immunity (PTI; Thomma et al., 2011; Zou et al., 2012; Achor et al., 2020; Andrade et al., 2020). In this way, the morphological plasticity of *Ca*L spp. may represent an advantage, allowing adaptation in either the citrus phloem sap or in the ACP. Bacterial or host factors that modulate these dynamic morphological changes are yet unknown.

*C*Las, like other plant-pathogenic bacteria, is believed to secrete effectors (virulence factors) into the cytoplasm of the hosts. The action of these effectors can create favorable environments for colonization and proliferation (Toruño et al., 2016). The expression of effectors needs to be coordinated spatio-temporally to allow the bacteria to thrive and shift between different lifestyles (plant and psyllid). The small *C*Las genome (~1.2 Mb) only harbors genes for the general protein secretory (Sec) pathway and the Type I secretion system (T1SS) for translocating effectors out of the cell (Duan et al., 2009; Li et al., 2012). Several Sec secreted-dependent effectors (SDEs) have been identified by bioinformatic analysis (Pitino et al., 2016; Clark et al., 2018; Shi et al., 2019; Ying et al., 2019). Although their transgenic expression in model and citrus plants have suggested roles in plant defense modulation, whether they are actually secreted to the extracellular environment is still a matter for discussion (Prasad et al., 2016; Clark et al., 2020; Pang et al., 2020; Du et al., 2021).

The *C*Las T1SS machinery, similar to other bacterial T1SSs, is composed of three proteins: an inner membrane ATP-binding cassette (ABC) transporter (PrtD), a transmembrane protein (HlyD), and an outer membrane export protein (TolC; Duan et al., 2009; Li et al., 2012; Thapa et al., 2020). The ABC transporters of bacterial T1SSs often show high specificity in binding their unfolded substrates that are translocated to the extracellular side of outer membrane, mostly in a single-step secretion strategy, without periplasmic intermediate (Linhartová et al., 2010; Baumann, 2019; Spitz et al., 2019; Hui et al., 2021). However, the amenability of the T1SS to secrete substrates of different nature and structure has hampered the identification of a canonical -terminal secretion signal.

One of the putative T1SS substrate identified in the *C*Las genome is CLIBASIA_01345, a protein with similarity to members of serralysin-type metalloproteases (Cong et al., 2012; Li et al., 2012). Among the best-characterized secreted metalloproteases is PrtA – also named serralysin or PrtS – from *S. marcescens*. PrtA plays a key role as bacterial virulence factor, it is involved in cytotoxicity and modulates the immune responses of its host (Lee et al., 2017). *In vitro* studies show that PrtA inhibits the attachment of insect hemocytes and mammal macrophages to tissue surfaces by degrading adhesive molecules (Ishii et al., 2014). Moreover, PrtA contributes to the ability of *S. marcescens* to develop a mature biofilm which could facilitate colonization and host invasion processes (Bruna et al., 2018). In another example, AprA, an extracellular alkaline metalloprotease from *Pseudomonas syringae*, mediates the degradation of flagellin monomers, leading to the evasion of the first layer of immune responses from *Arabidopsis thaliana* and tomato (Pel et al., 2014).

The function of CLIBASIA_01345 (hereafter Las1345) in *C*Las and its contribution to host-*C*Las interaction is unknown, although its higher expression in the citrus phloem (5.5-fold) as compared to the psyllid vector (Yan et al., 2013) suggests that *C*Las uses this protein to modulate its lifestyle in the two hosts. *C*Las as well other pathogenic *Ca*L species remain unculturable in artificial media, making the use of surrogate bacterial models the only available approach to analyze putative gene function associated with *C*Las pathogenesis (Vahling-Armstrong et al., 2012; Kuykendall et al., 2012b; Pagliai et al., 2014; Barnett et al., 2019; Jain et al., 2019). Here we study the role of Las1345 as a potential virulence factor associated with bacterial adaptation to different hosts. *Rhizobium leguminosarum* and *S. marcescens*, as well as the phytopathogen *Xanthomonas campestris* pv. *campestris* (*Xcc*) were used as surrogate models to study Las1345 function in bacteria, whereas the role of Las1345 in planta was investigated by transient expression in model plants.

## Materials and methods

### Sequence data acquisition and multiple sequence alignment

The genome sequences of the three citrus-infecting *Ca*L species [Las (CP001677.5), *C*Laf (CP004021.1) and *C*Lam (CP006604)] are available at the National Center for Biotechnology Information (NCBI) database. Protein sequences, based on the homology to Las1345, were downloaded from NCBI under the following accession numbers: serralysin-like proteins from *C*Las strain psy62 (WP_015452346), from *C*Laf strain PTSAPSY (AKK19938.1) and from *C*Lso strain ZC1 haplotype B (WP_013461860); PrtA from *S. marcescens* (CAA39139.1), Ser1 from *S. liquefaciens* FK01 (BAK39731) and PrtC from *Dicheya chrysanthemii* B374 (WP_038909783.1). The sequences were aligned using Clustal Omega and MultiAlin (Corpet, 1988; Sievers and Higgins, 2018), following a manual adjustment in the C-terminal sequence, considering the sequence pattern associated with the ABC exporter motif. Las1345 was homology modeled using Swiss Model server (Waterhouse et al., 2018).

### Bacterial strains, grow conditions and cloning

Bacterial strains and plasmids are described in Table 1. *Rhizobium leguminosarum* bv. *viciae* A34 (*A34*), *S. marcescens* mutant in *prtA* (*prtA*), *Xanthomonas campestris* pv. *campestris* (*Xcc*) wild type and the *gumB* mutant strains were grown in tryptone-yeast (TY), Miller’s Luria-Bertani (LB) and peptone-yeast-malt (PYM) medium, respectively (Russo et al., 2006; Torres et al., 2007; Bruna et al., 2018). Plasmids were mobilized into *R. leguminosarum* bv. *viciae* A34 and *S. marcescens* by biparental mating using *Escherichia coli* S17 cultured LB, at 37°C (Vozza et al., 2016; Bruna et al., 2018). Conjugation was performed at 28°C and 37°C for *R. leguminosarum* and *S. marcescens*, respectively. *Xcc* was transformed by electroporation (Rigano et al., 2007). Bacterial growth was monitored at an optical density of 600 nm (OD600).

**Table 1:** Bacterial strains and plasmids.

Strain or plasmid	Description	Source or reference
Strain		
*R. leguminosarum* A34	*R. leguminosarum* 8401 derivatives carrying pSym plasmid Prl1ji, Str^r^	Russo et al. (2006)
*prtA*	*S. marcescens* RM66262; *prtA*::pKNOCK, Cm^r^	Bruna et al. (2018)
*prtA/PrtA*	*S. marcescens prtA;* pBBR2::*prtA,* Kan^r^	Bruna et al. (2018)
*Xcc*	*X. campestris* pv*. campestris* 8,004, Rif^r^	Daniels et al. (1984)
*gumB*	*X. campestris* pv. *campestris* strain 8,397, [*X. campestris* pv. *campestris* 8,004 *gumB*::Tn5, Kan^r^]	Vojnov et al. (1998)
*E. coli S17*	*E. coli [thiJ thr leu tonA lacY 61lic recA*::RP4-2-Tc::Mu lpir] Km^r^	Russo et al. (2006)
*E. coli* ArticExpress (DE3) RIL	*E. coli B* F^−^ *ompT hsdS*(rB– mB–) dcm^+^ Tet^r^ gal λ(DE3) *end*A Hte [*cpn*10 *cpn*60 Gent^r^] [argU ileY leuW Str^r^]	Agilent technologies
*A. tumefaciens*	*A. tumefaciens* GV3101, Rif^r^, Gm^r^	
Plasmid		
pBBR2	pBBR1-MCS2, Km^r^, broad range	Kovach et al. (1995)
pBBR2::Las1345	pBBR1-MCS2::CLIBASIA01345, Km^r^	This work
pBBR2::HisLas1345	pBBR1-MCS2::His_x6_CLIBASIA01345, Km^r^	This work
pMP2444	pBBR1-MCS5::GFP, Gm^r^	Rigano et al. (2007)
p15TV-L	Expression vector, His_x6_, Amp^r^	Pagliai et al. (2010)
p15::HisLas1345	p15TV-L::CLIBASIA01345, Amp^r^	This work
pMDC83	Binary vector, 2x35S promoter*,* C-terminal GFP*,* Kan^r^	Curtis and Grossniklaus (2003)
pMDC83::Las1345-GFP	35S::CLIBASIA01345-GFP, Kan^r^	This work
pMDC83-GFP	pMDC83 *ccd*^−^, Kan^r^, allow 35S::GFP expression	This work
pEarlyGate100	Binary vector, 2x35S promoter, Kan^r^	Earley et al. (2006)
pEarlyGate100::Las1345	35S::CLIBASIA01345, Kan^r^	This work

*CLIBASIA_01345* was amplified from *C*Las-infected plant tissue *via* polymerase chain reaction (PCR) and subsequently cloned into p15TV-L (Pagliai et al., 2010) to obtain p15::HisLas1345. This vector was used as template to obtain pBBR2::HisLas1345 by restriction sites cut with *Nco*I and *Hind*III. The plasmid p15::HisLas1345 was also used as template to amplify tagless *CLIBASIA_013145* to generate pBBR2::Las1345 using the following primers: 5-GTCGGTACCATGCATAATATAAAACCGG-3/5-GTCAAGCTTTCAGGAAAAATCATGATTTA-3. To express Las1345 in plants, *CLIBASIA_01345* was amplified from p15:HisLas1345, adding *Kpn*IATG/*Hind*III sites with the following primers 5-GTCGGTACCATGCATAATATAAAACCGG-3 and 5-GTCAAGCTTTCAGGAAAAATCATTTA-3 and cloned into pENTR3c-Las1345 to generate pENTR3c::Las1345. This vector was amplified with AHL primers (5-TAGTTAGTTACTTAAGCTCGGGC-3/5-CAGAGCTGCAGCTGGATGGC-3) and the PCR product was transferred into binary vectors (pMDC83 and pEarlyGate100) using Gateway LR Clonase II following manufacturer instructions (Thermo Fisher Scientific, Waltham, United States).

### Las1345 expression and purification

The recombinant plasmid p15::HisLas1345 was used to transform *E. coli* ArticExpress competent cells for further expression and purification. Cells were grown at 30°C until OD_600_ ~ 0.7. Induction was made using 0.5 mM IPTG and 6-h incubation at 19°C. The cell pellet was suspended in binding buffer (50 mM HEPES pH 7.5, 250 mM NaCl, 5% (v/v) glycerol, 2.5 mM TCEP and 5 mM Imidazol). The thawed cells were passed through a French Press (French pressure cell press 40 K, Thermo Scientific). Three runs at 800 pressure gauge (Phcp) were conducted for each sample. Samples were released at 15 drops/min. The clarified lysed was purified using Ni-NTA resin (Qiagen, Germantown, United States) as described previously (Pagliai et al., 2014). Purified protein was dialyzed against 10 mM HEPES pH 7.5, 50 mM NaCl, 10 mM MgCl_2_, 2 mM CaCl_2_, 5% (v/v) glycerol and 0.25 mM TCEP. Protein was concentrated using Vivaspin centrifugal concentrators (Sartorius, Bohemia, United States) treated with Triton X-100 and the purity was analyzed in gel. The purified protein was refolded by dilution into cold buffer (50 mM Tris, 50 mM NaCl, 0–0.2 mM CaCl_2_, pH 7.8) on ice for 20 min prior to activity. The proteolytic activity of the purified protein (pHisLas1345) was evaluated under different amount of protein and the addition of divalent cations (Zn^2+^, Co^2+^, Cu^2+^ and Ca^2+^) in the chloride salt form (Supplementary Figure 1).

### Bacterial cell fractionation

For analysis of secreted and intracellular proteins, bacteria (*R. leguminosarum*, *S. marcescens* and *Xcc*) were grown for 48 h at 28°C in TY, LB or PYM medium, respectively to an OD_600_ ~ 0.6. The cell culture (3 ml) was centrifuged at 6,500 rpm and both the extracellular medium (supernatant) and the cellular precipitate were reserved for protein extraction. Supernatant (2 ml) was clarified with a 0.2 μm-syringe filter to obtained a cell free supernatant fraction (SF). Extracellular proteins were concentrated from the SF by precipitation with 10% (v/v) trichloroacetic acid (Merck, Darmstadt, Germany) and incubated on ice for 2 h. Proteins were recovered by centrifugation (12,000 rpm, 30 min) and washed twice with 500 μl acetone (Cicarelli, Santa Fe, Argentina). The precipitate was dried and the proteins were solubilized in the proper buffer for activity or immunodetection. Intracellular proteins were recovered from the soluble and insoluble cell fractions (CFs and CF_I,_ respectively) prepared from cellular precipitate. Cells were washed twice with 10 mM Tris, 5 mM EDTA pH 8 and lysed by sonication at a frequency of 20 kHz. The lysate was centrifuged (12,000 rpm, 30 min) at 4°C to recovered the pellet (CF_I_) and the supernatant (CFs) fractions. Proteins in the CFs were precipitated with acetone (Cicarelli, Santa Fe, Argentina) on ice for 2 h and recovered by centrifugation (12,000 rpm, 30 min). CFs and CF_I_ pellets were resuspended in the proper buffer for activity or immunodetection.

### Immunodetection

SF, CFs and CF_I_ pellets were resuspended in loading buffer [60 mM Tris, 10% (v/v) glycerol, 180 mM β-mercaptoethanol, 0.003% (w/v) bromophenol blue and 2% (w/v) SDS, pH 6.8], separated by 12% sodium dodecylsulfate-polyacrylamide gel electrophoresis (SDS-PAGE) and visualized by staining with Coomassie brilliant blue R-250 (Genbiotech, Buenos Aires, Argentina). Western blot was carried out using standard techniques. Briefly, after SDS-PAGE, proteins were transferred onto pre-wetted polyvinylidene difluoride membranes (PVDF- Immun-BlotV^R^, BioRad, CA, United States). Immunodetection was performed using polyclonal antibodies against His tag (1:6,000 dilution, #SAB1306085 Sigma, Merck, Darmstadt, Germany), *Rhizobium* adhering-protein A1 (RapA1; 1:5,000 dilution; Vozza et al., 2016) or *E. coli* Glucose-6-phosphate dehydrogenase (G6PD; 1:500 dilution; Giró et al., 2006) and then revealed using a peroxidase-conjugated goat anti-rabbit IgG (H + L; 1:3,000 dilution, #1706515, BioRad, Des Plaine, IL, United States) and Pierce™ ECL Western Blotting Substrate (Thermo Scientific, Petaluma, CA, United States) according to the manufacturers. Chemiluminescence was detected using an CCD camera (ChemiDoc™ XRS^+^, BioRad, Des Plaine, IL, United States). For probe stripping and rehybridization, the filters were incubated with stripping buffer (0.05 M Tris, 1% (w/v) SDS, 0.8% β-mercaptoethanol, pH 6.8) for 45 min at 30°C and washed five times with TBS (20 mM Tris, 150 mM NaCl, pH 7.6) supplemented with 0.05% (v/v) Tween (Promega, Fitchburg, WI, United States).

### Plant growth and pathogenicity assays

*Arabidopsis thaliana* and *Nicotiana benthamiana* were grown under controlled conditions in a growth chamber with a temperature of 25°C–27°C and a photoperiod of 16 h light/8 h dark and light intensity of 150–200 μE/s m^2^. All plant inoculations involved a minimum of 10 plants per strain tested and one leaf per plant was inoculated. Bacterial suspensions of *Xcc* (10^7^ CFU/ml in 10 mM MgCl_2_) were inoculated by pressure infiltration (Siciliano et al., 2006). Disease progression (water-soaking and necrosis) was monitored phenotypically and by bacterial growth curves. Bacterial population was determined in five samples according to the method described by Rigano et al. (2007). Each sample was obtained from three leaf disks of 1 cm^2^ collected randomly from different inoculated leaves. Symptom development were assayed by conductivity measurements using leaf disks submerged in milli-Q water following protocols previously described (Chiesa et al., 2019). Six samples were measured, each one obtained from three leaf disks of 1 cm^2^ collected randomly from 10 inoculated leaves. Photosystem II quantum efficiency (ϕPSII) was measurements at 25°C on one dark-adapted leaf from 15 plants using MultispeQ V 2.0 (PhotosynQ INC, East Lansing, MI, United States). These assays were repeated three times.

### *Agrobacterium*-mediated transient expression and protein localization

To evaluate the function of Las1345 in plants, *N. benthamiana* leaves were agroinfiltrated with *A. tumefaciens* GV3101 transformed with the corresponding plasmid (Table 1), to overexpress Las1345 with and without a GFP tag. Molecular techniques and agroinfiltration were performed as described previously (Sánchez et al., 2010; Enrique et al., 2011). GFP versions were analyzed to study protein localization by confocal laser scanning microscopy (CLSM). Nucleus and membrane were marked with DAPI (Thermo Fisher Scientific, Waltham, United States) and FM 4-64 (Sigma, Merck, Darmstadt, Germany), respectively. Images were taken 2 days after agroinfiltration with a Zeiss LSM 880 confocal laser scanning microscope (Carl Zeiss Microscopy GmBH, Jena, Germany) using the following parameters, GFP (488/527 nm), FM 4-64 (515/604 nm) and DAPI (405/449 nm). For CLSM analysis, each construct was infiltrated into two leaves of 10 plants.

For plant protein extraction, six leaf disks (1 cm^2^) collected randomly from different agroinfiltrated plants were pulverized in pre-chilled pestle and mortar using liquid nitrogen and the powder was resuspended in 0.5 ml of extraction buffer (50 mM Tris, 100 mM KCl, 10% (v/v) glycerol, pH 7.5). Supernatant was recovered after centrifugation (12,000 rpm, 20 min) at 4°C and kept at −80 for protease activity measurements.

### Protease assays

Protease activity was assayed using milk or azocasein (#A2765, Sigma, Merck, Darmstadt, Germany) as substrates as described previously (Bruna et al., 2018). For qualitative analysis, the samples were inoculated on LB agar plates supplemented with skim milk at 2% (w/v) and incubated for 16 h at 30°C. Distinct clearing of the milk around the colony was used as a protease activity indicator. For quantitative analysis, protease activity was measured using azocasein as substrate. Samples of SF, CF_S_ and CF_I_ (50 μl) were mixed with 10 μl of 1% (w/v) azocasein and 140 μl of phosphate-buffered saline (PBS) and incubated for 1 h at 37°C. The reaction was stopped by addition of 80 μl of 10% (v/v) trichloroacetic acid, and the mix was incubated on ice for 15 min. Protease activity was repeated five times using six replicates of each strain. The same protocol was used to evaluate protease activity with both the purified HisLas1345 protein from *E. coli* and plant protein extracts. For these analyzes, measurements were normalized by protein quantification using the Bradford assay kit (BioRad, Des Plaine, IL, United States) and repeated three times.

### Cell motility and production of the extracellular polysaccharide (EPS) xanthan

Swimming, swarming and sliding motility assays were carried out as previously described (Malamud et al., 2011, 2013). Briefly, overnight cultures were normalized to an OD_600_ ~ 0.8 and 3 μl were used to inoculate 0.25% (w/v) agar NYGB medium plates (swimming) or 0.5% (w/v) agar NYGB medium (sliding/swarming). Plates were incubated 72 h at 28°C. Motility was assessed quantitatively by measure the circular halo formed by the growing bacterial cells in 10 different culture plates. This assay was repeated three times.

Exopolysaccharide (EPS) xanthan quantification was performed as described previously (Vojnov et al., 1998). To measure EPS xanthan production, strains were grown in PYM medium supplemented with 1% (w/v) D-glucose at 28°C for 24 h with shaking (156 rpm) in 250-mL flask. EPS xanthan was precipitated from culture supernatants using 2 vol of ethanol. The precipitated EPS xanthan was collected, washed with ethanol, dried and weighed. Each assay included eight to 10 replicas and the experiment was repeated three times.

### Biofilm analysis by CLSM

To analyze biofilm structure, GFP-expressing *Xcc* cells were transformed with either pBBR2::Las1345 or pBBR2 as the empty vector control. Both strains were grown at 28°C on PYM medium supplemented with 50 μg/ml kanamycin. Cultures were diluted in Y minimal medium (YMM) to reach OD_600_ ~ 0.02 and aliquots of 500 μl were transferred to 8-wells chambers containing a 1-mm thick borosilicate glass (Nunc, Wiesbaden, Germany), as described by Rigano et al. (2007). Strains were incubated for 4 days at 28°C. Congo red (10 μM) was added to YMM to visualize extracellular components, such as exopolysaccharides, curli amyloid proteins and adhesins which conform the biofilm matrix (Serra and Hengge, 2017). Congo red staining has been used to detect amyloid fibers in *Xanthomonas* spp. (Oh et al., 2007; Kraiselburd et al., 2012). Biofilm formation was monitored using a Zeiss LSM880 confocal laser scanning microscope (Carl Zeiss Microscopy GmBH, Jena, Germany) by excitation at 488 nm with the argon laser line and 20x NA = 0.8 Plan-Aphocromat objective (Carl Zeiss Microscopy GmBH, Jena, Germany). To analyze the tridimensional structure of the biofilm, the XY area was screened in 35 Z-intervals (Z-stack) automatically captured up to a 25.5 μm range at green (488/520 nm, pinhole 0.84 A.U.) and red (543/619 nm, pinhole 0.70 A.U.) channels, respectively. Biofilm images were obtained by ZEN BLACK software (Carl Zeiss Microscopy GmBH, Jena, Germany) and analyzed with COMSTAT2 (Heydorn et al., 2000; Vorregaard, 2008).^1^ Each assay includes triplicates (3 wells per strain) and it was repeated three times.

### Bacterial RNA extraction, reverse transcription, and quantitative PCR (qPCR)

The expression levels of three (*filE*, *fliF* and *flgL*) flagellum assembly-related genes of *Xcc* were analyzed by qPCR. *Xcc*/Las1345 and *Xcc*/pBBR2 cells were grown in YMM at 28°C to reach an OD_600_ ~ 0.8. The cells (10 ml) were collected and total RNA was extracted using TRIzol Reagent (Thermo Fisher Scientific, Walthman, United States), following treatment with RNase-free DNase (Promega, Wisconsin, United States). Reverse transcription was performed using RevertAid reverse transcriptase (ThermoFisher Scientific, Carlsbad, United States), 2 μg of DNase-treated total RNA and 0.5 μg/μl random hexamers, according with the manufacturer’s instructions. cDNAs were used for qPCR using Hot FIREPol^®^ EvaGreen qPCR mix plus (Solis BioDyne, Tartu, Estonia) and 0.25 μM of each primer. Quantitative PCRs (qPCR) were performed for 42 cycles according to the following conditions: denaturation at 95°C for 15 s, annealing at 60°C for 30 s and extension at 72°C for 40 s. After amplification, melting curves were performed to exclude artifactual amplifications. The *16S* ribosomal RNA was used as internal control. Primers were designed based on the *Xcc* 8004 (CP029484.1) genome sequence. Flagellar genes were amplified with the following primers, *fliE*_fw 5′-AGCTTCAGCGAGACCTTGCG-3′ and *fliE*_rv 5′-CAGATCGGCACTGGGGTCAC-3′; *fliF*_fw 5′-AAGTTCCAAGAGCGCCACCC-3′ and *fliF*_rv 5′- ACGATCTTGCCCTTGGCACC-3′; *flgL_*fw 5′- ATGGCAATGCGCCTTTCATC and flgL_rv 5′- GCGGATGCGCATGAAGATTT-3′; *16S_*fw 5′-AGGACCTTCGGGCTT-3′ and 5′-TGTCTCAGTTCCAGTG-3′ (Yan et al., 2019).

### Statistical analysis

Data were analyzed according to Student’s *t*-test through InfoStat Software v2017 (Di Rienzo et al., 2017), excepting protease activity in bacterial cell fractions and EPS production data that were subjected to one-way analysis of variance (ANOVA). Two-way ANOVA was used to analyze the data from protease activity in plant crude extract. In both cases, means were analyzed using Tukey’s test *p* < 0.05).

## Results

### Structural analysis of Las1345 reveals absence of calcium-binding GG repeats in the RTX domains

**Figure 1:**
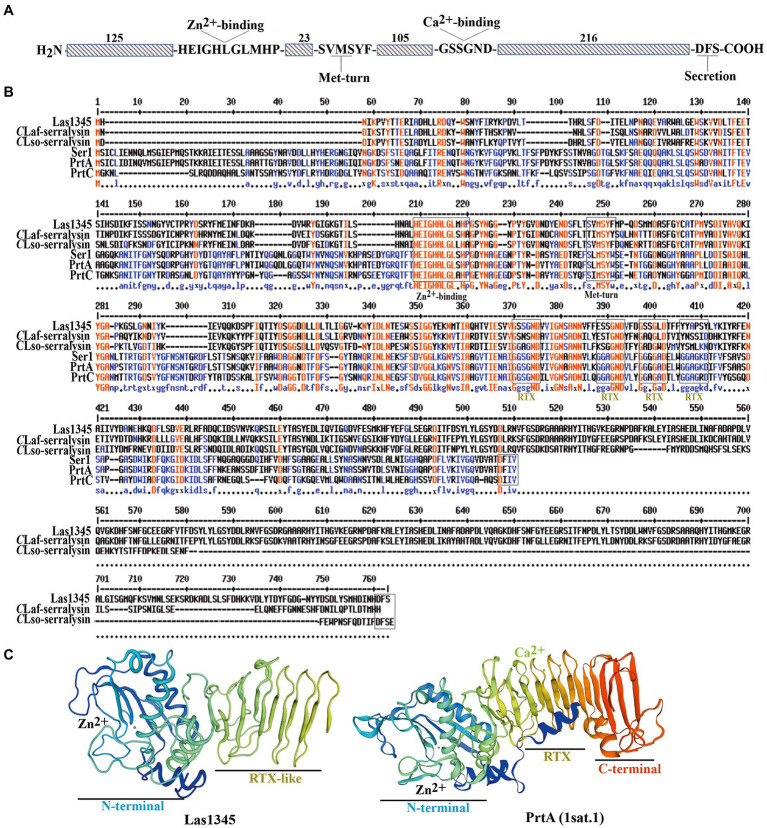
Multiple sequence alignment and representative tertiary structure of Las1345. **(A)** Schematic representation of Las1345 (CLIBASIA_01345). Conserved motifs associated with serralysin family protein are shown. HExxHxUGUxH, zinc-binding motif; SxMxY, a Met-turn motif; GGxGxDxUx, calcium-binding motif (glycine-rich repeats) where U represents an aliphatic amino acid and X can be any residue; Dxxx, T1SS secretion signal. **(B)** Sequence alignment of the serralysin-like proteins encoded by *Candidatus* Liberibacter (*Ca*L) asiaticus (*C*Las) strain psy62 (Las1345, WP_015452346); *Ca*L africanum strain PTSAPSY (*C*Laf_Serralysin, AKK19938.1); *Ca*L solanacearum strain ZC1 (*C*LsoB_Serralysin, WP_013461860); *Serratia marcescens* (PrtA, CAA39139.1); *S. liquefaciens* strain FK01 (Ser1, BAK39731) and *Dickeya chrisantemy* strain B374 (PrtC; PDB 1K7I_A). Alignment was obtained by ClustalW and Multaline. Low consensus alignment (50%) is shown in blue letters while high consensus alignment (90%) is shown in red. Protein domains were identified by Pfam. Protein domains, showing the metalloprotease sequence (HExxHxxGxxHP) motif, the met-turn (SxMSYF/W) motif, the four RTX repeats (the classic, GGxGxD and the divergent, GSxGxD) and the ABC exporter motif (Dxxx) are indicated in boxes. **(C)** Tertiary structure of Las1345 domains based on a homology model derived from PrtA structure (PBD ID 1sat.1; Baumann, 1994; QMEANG 0.65 ± 0.5). The N terminal Zn-binding domain and the RTX β-rolls are colored in rainbow. Zinc and calcium ions are represented by gray and green spheres, respectively.

Genome comparison between *C*Las and *Ca*L solanacearum (*C*Lso, causing zebra chip disease in potato) revealed that the *Ca*L serralysin-like gene (Figure 1A) is largely conserved in terms of size and localization, being next to a gene cluster encoding the T1SS (Li et al., 2012; Ravindran et al., 2018). In this work we have included sequence comparison with the other two HLB-associated *Ca*L species, africanus strain PTSAPY (*C*Laf) and americanus strain São Paulo (*C*Lam; Wulff et al., 2014; Lin et al., 2015). A putative serralysin gene was found in *C*Laf genome (Figure 1B). Comparative protein sequence analysis indicates that Las1345 (665 amino acids, aas) has 63.77 and 65.67% amino acid identity with the serralysin-like protein of *C*LsoB (577 aas) and *C*Laf (642 aas), respectively. These two proteins are longer than the characterized serralysins from *Serratia* spp. (PrtA and Ser1, 504 aas each) and *Dickeya chrysanthemi* (former *Erwinia*; PrtC, 479 aas; Figure 1B). Las1345 share 30.41%, 31.23%, and 31.30% amino acid identity with PrtA, Ser1 and PrtC, respectively. Las1345 conserves the N-terminal metzincin-type metalloprotease motif HExxHxxGxxHP, including the last proline, characteristic for serralysins. The three histidines in this motif are zinc ligands and the glutamic acid serves as catalytic base (Baumann, 1994, 2019). Twenty-three residues downstream from this motif, the conserved met-turn motif (SxMSYF/W) is found. Three-dimensional prediction shows that the N-terminal part of Las1345 has a folding topology similar to PrtA (Figure 1C). PrtA, Ser1 and PrtC have four conserved motifs (GGxGxDxUx) in the RTX (repeats-in-toxins) domain (Figure 1B). T1SS substrates that have these specific repeats belong to the RTX- protease family (Spitz et al., 2019; Hui et al., 2021). Three-dimensional structure of the RTX domain of PrtA shows that this domain consists of four parallel β-rolls, where two GG repeats coordinate one Ca^2+^ ion by interaction of the side chain of the aspartate residues and the carbonyl oxygens of the amino acids forming the repeat. Calcium ion binding is implicated in secretion and extracellular folding of the protein (Baumann, 2019; Spitz et al., 2019; Hui et al., 2021). Unlike PrtA, Las1345 has only one non-canonical RTX repeat, GSSGND, located between G_370_ and D_375_. It has been hypothesized that this consensus motif, instead of the typical nonapeptide repeat, may also be considered as a potential binding site for Ca^2+^ (Linhartová et al., 2010). Interestingly, the GSSGND motif in the RTX domain of Las1345 also folds in a right-handed β-roll (GSS) and connects a short strand as in PrtA. However, this RTX repeat is not recognized as a Ca^2+^ binding site by the modeling server (Figure 1C). Moreover, prior to the predicted non-cleaved C-terminal T1SS secretion signal (DFS) for serralysins, Las1345 exhibits a duplicated 84-residue sequence (Figure 1B).

These differences between Las1345 and characterized serralysins such as PrtA may influence Las1345 secretion *via* the T1SS and extracellular folding. However, the metzincin-type metalloprotease domain and the non-classical RTX repeats still supports the possibility of protease activity in Las1345.

### Las1345 has protease activity in the cytoplasm of *rhizobium leguminosarum* and *Serratia marcescens*

**Figure 2:**
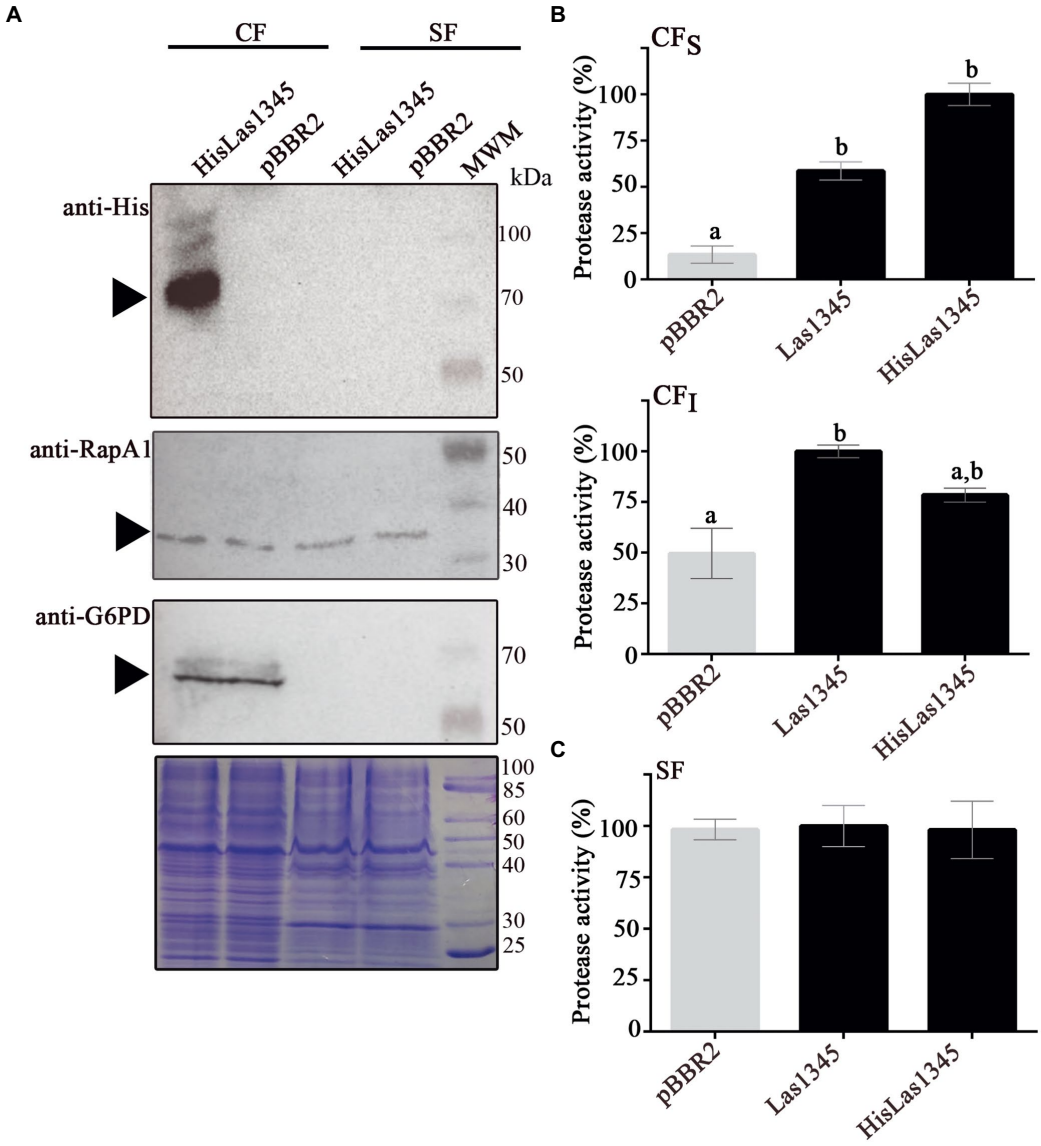
Las1345 has intracellular protease activity in *Rhizobium leguminosarum*. **(A)** Immunodetection of Las1345 (~75 kDa) in cellular (CF) and supernatant cell free fraction (SF) from *Rhizobium leguminosarum* A34 (*A34*) cells expressing Las1345, HisLas1345 or transformed with the empty vector (pBBR2), grown for 16 h at 30°C. *Rhizobium* adhering protein A1 (RapA1, ~36 kDa) and Glucose-6-phosphate dehydrogenase (G6PD, ~55 kDa) were used as extracellular and intracellular controls, respectively. **(B)** Protease activity in SF from *A34* cultures **(C)**, Protease activity in soluble and insoluble CF (CF_S_ and CF_I_) from *A34* cultures. The relative activity was expressed as the percentage of activity detected with respect to the maximum protease activity in the assay. Values are expressed as means ± standard deviations from six independent biological replicates. Different letters indicate significant differences at *p* < 0.05 (one-way analysis of variance, Tukey’s test).

*Ca*L is a member of the order *Rhizobiales* and it is phylogenetically related to the *Rhizobium-*type genus (Young et al., 2001; Duan et al., 2009; Kuykendall et al., 2012a). In order to study the putative secretion of Las1345, the symbiont *R. leguminosarum* was used as surrogate model, considering the T1SS similarity between *Ca*L *spp*. and *Rhizobium* (Duan et al., 2009; Kuykendall et al., 2012a). We first evaluated the putative secretion of Las1345 using cell fractionation in *R. leguminosarum* bv. *viciae* strain A34 (*A34*; Figure 2A). RapA1, an extracellular T1SS-secreted and surfaced associated protein from *R. leguminosarum* (Vozza et al., 2016) and the bacterial intracellular G6PD protein (Giró et al., 2006) were used as subcellular fractionation controls. An N-terminal His-tagged version of Las1345 (HisLas1345) was only detected in the cellular fraction of *R. leguminosarum*, suggesting either that the C-terminal domain of this protein is not recognized by the inner membrane ABC transporter of the T1SS of *R. leguminosarum* or that the N-terminal tag prevents Las1345 secretion to the extracellular medium (Figure 2A). Interestingly, accumulation of HisLas1345 in cellular fraction correlated with an increased protease activity, measured using azocasein as substrate (Figure 2B). To discard a tag-effect during the secretion process, an untagged version of Las1345 was expressed in *R. leguminosarum* and protease activity was measured in the supernatant fraction. In both tagged and untagged versions, Las1345 did not increase proteolytic activity in the supernatant fraction of *A34*, suggesting that Las1345 might not be secreted, at least in this surrogate system (Figure 2C). Moreover, in both versions of Las1345, an increase in proteolytic activity was detected both in the soluble and insoluble cellular fractions (CF_S_ and CF_I_). Interestingly, proteolysis was increased by more than 50% in CF_S_ of both *A34/*Las1345 (100 ± 3.15) and *A34/*HisLas1345 (78.45 ± 3.42) compared to the *A34*/pBBR2 (9.67 ± 2.33) and only 10%–20% in CF_I_ (Figure 2C).

**Figure 3:**
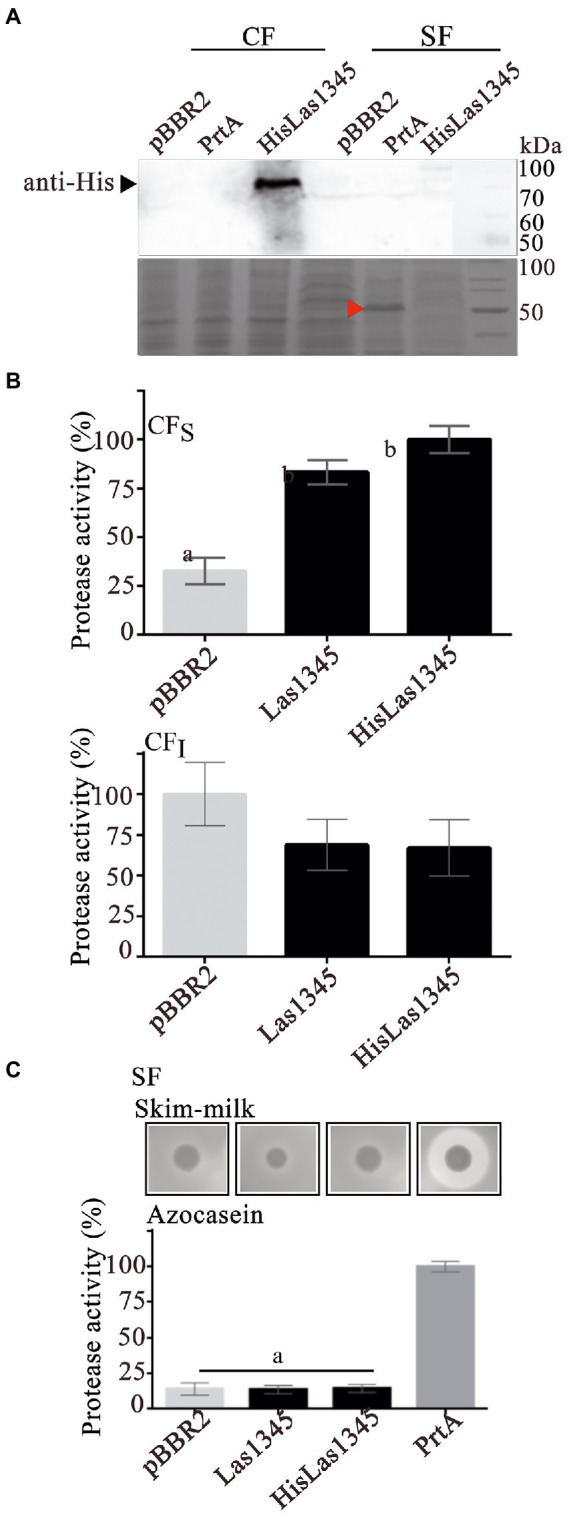
Las1345 protease activity in *Serratia marcescens*. **(A)** Immunodetection of Las1345 (~75 kDa) in cellular (CF) and supernatant cell free fraction (SF) *prtA* cells from *S. marcescens* expressing Las1345, HisLas1345 or transformed with an empty vector (pBBR2), grown for 16 h at 30°C. PrtA (~50 kDa) was visualized by Coomassie staining (red arrow) and used as control of extracellular secretion (SF). **(B)** Protease activity in soluble and insoluble CF (CF_S_ and CF_I_) from *prtA*/pBBR2, *prtA*/Las1345, *prtA*/FIGURE 3 (Continued)HisLas1345, and *prtA*/PrtA cells. The relative activity was expressed as the percentage of activity detected with respect to the maximum protease activity in the assay **(C)** Protease activity in SF from *prtA*/pBBR2, *prtA*/Las1345, *prtA*/HisLas1345, and *prtA*/PrtA cells was measured by detecting degradation of milk proteins (LB agar-skim milk plate), seen as a halo around the colony and by the azocaseinase assay. Values are expressed as means ± standard deviations from six independent biological replicates. Different letters indicate significant differences at *p* < 0.05 (one-way analysis of variance, Tukey’s test).

As shown in Figure 1, PrtA from *S. marcescens* shows homology with Las1345, mainly in the catalytic domain. Therefore, a *S. marcescens prtA* mutant strain (*prtA*) was transformed with pBBR2::HisLas1345 and pBBR2::Las1345 to evaluate Las1345 secretion and its proteolytic activity in this surrogate model. *prtA* complemented by *trans* expression of *prtA* from the pBBR2::*prtA* plasmid or transformed with pBBR2 were used as positive and negative controls, respectively. Similarly to the findings observed in *Rhizobium*, HisLas1345 was only detected in the cellular fraction of *prtA* (Figure 3A) in contrast with the positive control (PrtA) which was detected in the supernatant fraction (Figure 3A). As expected, based on the cellular localization, protease activity using azocasein as substrate was only detected in the CF_S_ fractions of *prtA*/HisLas1345- and *prtA/*Las1345, increasing the proteolytic activity as compared to the control (pBBR2; Figure 3B). The absence of protease activity in the SF of *prtA*/Las1345 and *prtA*/HisLas1345 (Figure 3C) reinforces the results obtained for the serralysin-like protein of *C*Lso in *S. liquefaciens* (Ravindran et al., 2018). Proteolytic activity of HisLas1345 was also confirmed using the purified protein (pHisLas1345) from *E. coli* (Supplementary Figure 1).

Taken together, these results indicate that Las1345 is not secreted by bacteria phylogenetically related to *C*Las, such as *R. leguminosarum*, nor by another surrogate bacterium such as *S. marcescens*. However, this protein shows proteolytic activity in the soluble cell fraction when expressed in these surrogates. We hypothesize that the long C-terminal region and the absence of multiple calcium-binding domains in the RTX motif of Las1345 allows this protein to be in its active conformation within the cell but hinders secretion to the extracellular space.

### Las1345 does not compromise plant cell integrity

**Figure 4:**
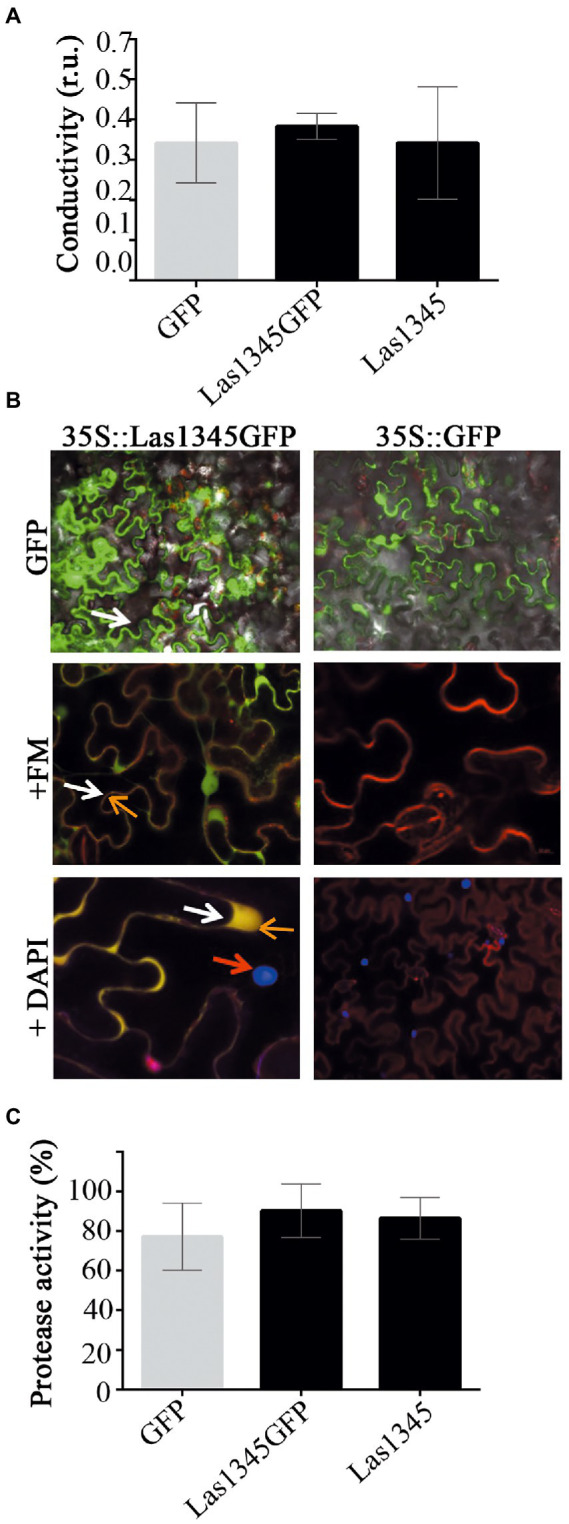
Las1345 expression in plant cells does not affect cell integrity. **(A)** Quantification of cell death in *N. benthamiana* leaves overexpressing GFP, Las1345-GFP, or Las1345 by measurement of conductivity at 3 days post agroinfiltration (dpi). Values are expressed as mean ± standard deviation of six samples. Each sample was obtained from three leaf disks of 1 cm^2^ collected randomly from different agroinfiltrated leaves. Data was analyzed by two-way analysis of variance and Tukey’s test at *p* < 0.05. Each(Continued)FIGURE 4 (Continued)assay was repeated three times. Scale bar, 10 μM. **(B)** *N. benthamiana* leaves expressing Las1345-GFP (green, white arrow) or GFP were imaged by confocal microscopy at 2 days post infiltration (dpi). FM4-64 (FM) and DAPI were used to distinguish membrane (red, orange arrow) and nucleus (blue, red arrow), respectively. Co-localization of GFP and FM is shown in yellow. **(C)** Protease activity using azocasein as substrate in total plant protein extracts (50 μg) from *N. benthamiana* leaves overexpressing GFP, Las1345GFP or Las1345 at 2 dpi. Activity is expressed as percentage relative to the highest activity value obtained among samples. Values are expressed as mean ± standard deviation of three samples. Each sample contains six disks from different agroinfiltrated leaves. Data was analyzed by two-way analysis of variance and Tukey’s test at *p* < 0.05.

Las1345 secretion to the extracellular medium could not be detected in surrogate bacterial models. However, this does not rule out Las1345 secretion in *C*Las *via* other non-conserved secretion pathways, or the putative role of Las1345 inside the plant cell. Phenotype changes have been observed *via* ectopic expression of multiple Sec secreted-dependent effectors *in planta*, even though the secretion of these proteins have not been demonstrated (Pitino et al., 2016; Clark et al., 2020; Pang et al., 2020; Du et al., 2021). Additionally, several extracellular metalloproteases from phytopathogenic bacteria, including AprA and Prt2/3, take part in modulating the plant immune defense, particularly through processes associated with plant cell wall degradation (Dow et al., 1990, 1993; Pel et al., 2014). Thus, observing whether Las1345 affects or not the plant cell in the hypothetical case that it could be secreted, could help to gain insight into the real scenario where Las1345 is functional. To explore the effect that Las1345 expression could eventually have in plant tissues, *N. benthamiana* leaves were infiltrated with an *Agrobacterium* culture harboring 35S-directed untagged and C-terminal tagged versions of Las1345. No phenotypic change between *N. benthamiana* plants expressing Las1345 constructs (35S::Las1345GFP or 35S::Las1345) and the control (35S,GFP) were observed. Moreover, plant cell integrity and conductivity were not affected by Las1345 expression *in planta* (Figure 4A), suggesting that cellular membrane damage is not taking place in *N. benthamiana* leaves when Las1345 is expressed. Fluorescence microscopy analyzes showed that Las1345GFP co-localizes with the plasma membrane dye FM 4–64, indicating that Las1345 is localized in these subcellular structures of the plant cell, particularly correlated with discrete cytosolic structures as a result of plasma membrane internalization (Figure 4B). Interestingly, no increase in protease activity was detected in Las1345-expressing tissues (Figure 4C). These results suggest that the intracellular environment in the leaf tissue is not favorable for the intrinsic proteolytic activity of Las1345.

### Las1345 affects cell motility and components of the extracellular matrix in the phytopathogen *Xanthomonas campestris* pv. *campestris*

**Figure 5:**
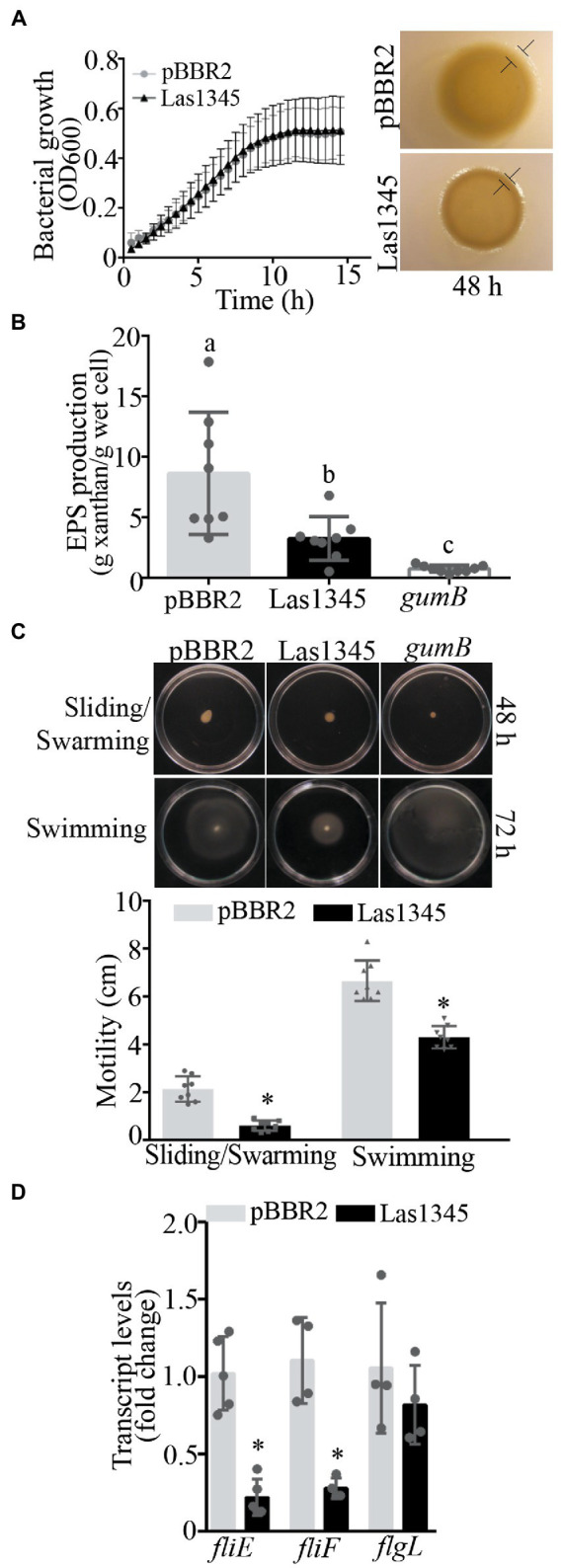
Las1345 expression reduce xanthan production and cell motility. **(A)**, *Xanthomonas campestris* pv*. campestris* (*Xcc*) growth in NYGB medium. Values are expressed as means ± standard deviation of five biological replicates. This assay was repeated five times. Macrocolony phenotype of Las1345-expressing *Xcc* and(Continued)FIGURE 5 (Continued)the control (*Xcc*/pBBR2) grown on 1% (w/v) agar NYGB medium at 28°C during 48 h. Differences at boundary zone are indicated with T-markers. **(B)** Quantification of xanthan secreted in Las1345-expressing *Xcc* cultures grown for 20 h at 28°C compared with control *Xcc/*pBBR2 and a xanthan deficient mutant (*gumB*). Values are expressed as means ± standard deviation of eight biological replicates. Different letters indicate significant differences at *p* < 0.05 (one-way analysis of variance, Tukey’s test). This assay was repeated three times. **(C)** *Xcc*/Las1345, *Xcc*/pBBR2 and *gumB* cultures were normalized by OD_600_ and inoculated on 0.5% (w/v) agar NYGB medium (sliding/swarming) and on 0.25% (w/v) agar NYGB medium (swimming). Pictures were taken after 48 or 72 h of incubatio at 28°C. Motility was measured as colony diameter (cm). Values are expressed as means ± standard deviation of 10 biological replicates. **(D)** Quantitative reverse transcription (qPCR) analysis of flagellum assembly-related genes (*fliE*, *fliF*, and *flgL*). Fold change of RNA levels, normalized to 16 s and relative to *Xcc*/pBBR2 is shown. Values are expressed as means ± standard deviation (SD) of five samples (replicates). Asterisks indicates significant differences between *Xcc*/Las1345 and *Xcc*/pBBR2 at ^*^p < 0.05 (Students *t*-test).

Although growth was similar between Las1345-expressing *R. leguminosarum* and *S. marcescens* and the same bacterial strains transformed with empty pBRR2 plasmids, morphological characteristics of colonies on the surface of agar-solidified media were different (Supplementary Figure 2). Differences in colony phenotypes were also observed when Las1345 was expressed into the phytopathogenic bacteria *Xcc*, whereas again both control and Las1354-expressing bacteria exhibit similar growth kinetics. Las1345-expressing *Xcc* developed dull and dry colonies with slightly thin edges, characteristic of defective cell motility, as compared with the smooth and thick-edges colonies found in the control (Figure 5A). This phenotype resembles that of *Xanthomonas* mutants deficient in the production of xanthan, the main EPS produced by xanthomonads, which affect the extracellular matrix and biofilm development (Yun et al., 2006; Rigano et al., 2007; Torres et al., 2007; Malamud et al., 2011). The *Xcc*/Las1345 colony phenotype, together with the fact that the proteolytic activity was also detected in the CF of *Xcc*/Las1345 (Supplementary Figure 3), lead to the hypothesis that the proteolytic activity may influence the assembly or the structure of the extracellular matrix. Accordingly, significant differences in EPS production were observed between *Xcc*/pBRR2 and *Xcc*/Las1345 (8.66 ± 3.04 g/g wet cell and 3.26 ± 1.81 g/g wet cell, *p* < 0.05) when grown in liquid medium (Figure 5B). EPS xanthan contribute to cell motility, cell-to-cell aggregation, bacterial fitness, and virulence in many different *Xanthomonas* species (Yun et al., 2006; Rigano et al., 2007; Torres et al., 2007; Malamud et al., 2011, 2013; Yaryura et al., 2015; An et al., 2020). This prompted us to study the effect(s) of Las1345 expression in the extracellular matrix of *Xcc*.

Bacterial motility is mainly controlled by the flagella which is responsible for swimming and swarming (Nakamura and Minamino, 2019). Another type of cell motility, sliding, is a flagella-independent type motility in which xanthan acts as a surfactant or surface-wetting agent to facilitate the movement of bacterial cells (Murray and Kazmierczak, 2008; Malamud et al., 2011). Therefore, we hypothesized that the differential macrocolony phenotype found between *Xcc*/Las1345 and *Xcc*/pBRR2 cells may be associated with differences in motility either associated with the flagella or with the formation of the extracellular matrix. Motility of *Xcc*/Las1345 was assayed in 0.25/0.5% (w/v) agar NYGB over a 2/3-day period. Sliding and swarming motility of *Xcc* was reduced in Las1345-expressing cells at 48 h (pBBR2 2.14 ± 0.53 cm vs. Las1345 0.61 ± 0.21 cm, *p* < 0.0001, Student’s *t*-test), in agreement with the reduced amount of EPS xanthan production compared with the *Xcc*/pBRR2 control (Figure 5C). A similar phenotype was observed in a *gumB* mutant of *Xanthomonas citri* subsp. *citri* (*X. citri*), as was reported by Malamud et al. (2011). Interestingly, *Xcc*/Las1345 also displayed very little swimming motility at 72 h (pBBR2 6.65 ± 0.0.84 cm vs. Las1345 4.31 ± 0.46 cm, *p* < 0.0001, Student’s *t*-test). However, *Xcc gumB* mutant showed a swimming motility similar to the wild-type (*Xcc*/pBRR2; Figure 5C).

In accordance with these results, the relative expression of flagellum assembly-related genes, such as *fliE* and *fliF* (Kan et al., 2018) was significant diminished in *Xcc*/Las1345 compared with control (*Xcc*/pBRR2). In contrast, the expression levels of *flgL* -associated with the hook structure- were similar between Las1345-expressing cells and the control (Figure 5D).

Taken together, all these results suggest that Las1345 expression influences the characteristics of the bacterial extracellular matrix by altering xanthan production, flagellum assembly and biofilm development.

### Las1345 interfere in cell adhesion, biofilm formation and the pathogenesis of *Xanthomonas campestris* pv. *campestris*

**Figure 6:**
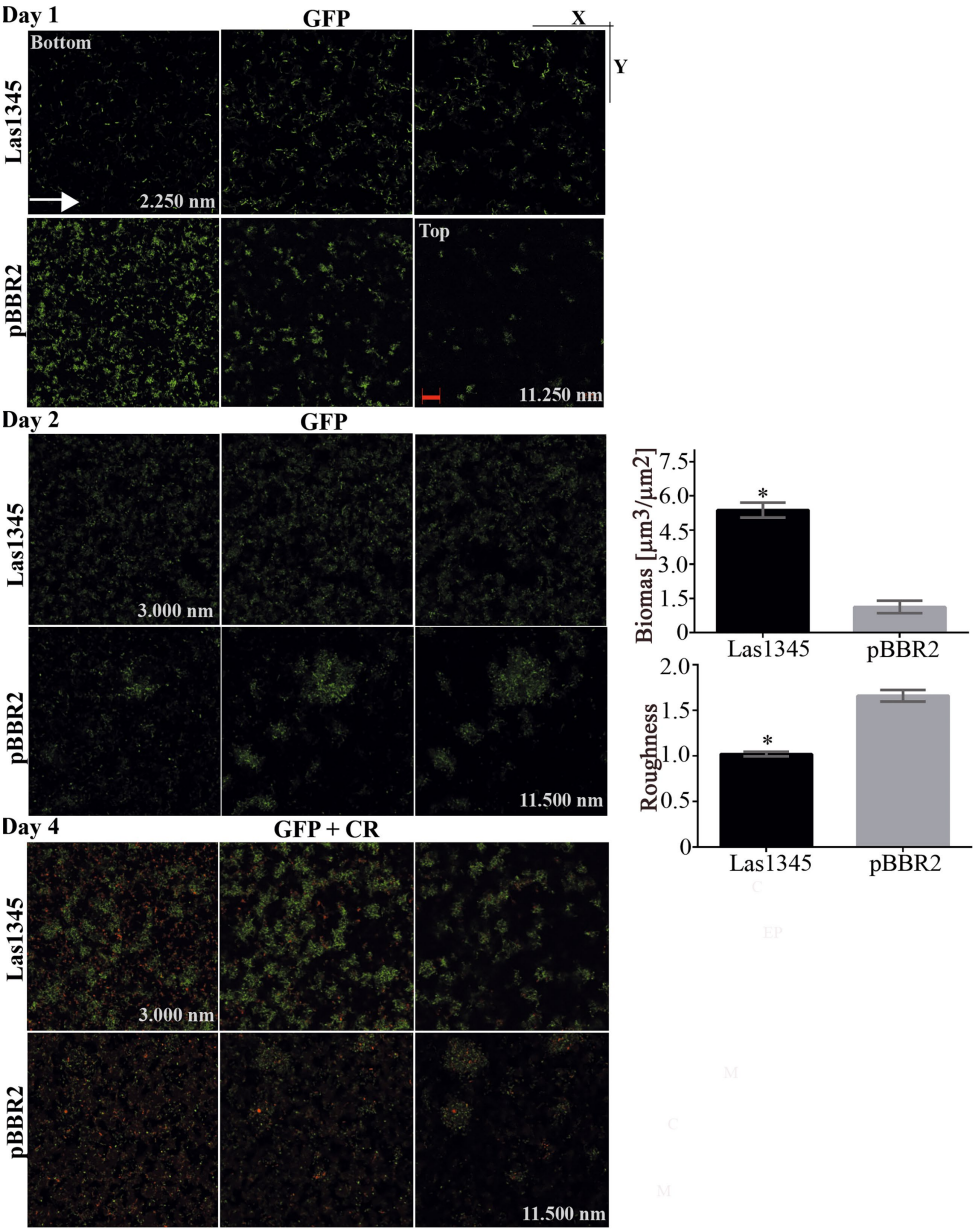
Biofilm architecture of *Xanthomonas campestris* pv*. campestris* (*Xcc*) is disrupted when Las1345 is expressed. GFP-labeled *Xcc* cells (*Xcc*-GFP) expressing Las1345 (*Xcc*-GFP/Las1345) were grown in 8-well chambers with a 1-mm thick borosilicate glass containing Y minimal medium and visualized at different stages of biofilm formation under confocal laser scanning microscopy for 4 days after inoculation at 28°C. Biofilm structure is shown as 2D-images of a single layer in the XY plane at different distances from the bottom well along the Z-axis (0–11898.21 nm) at day 1, 2 and 4. Green channel shows *Xcc*-GFP/Las1345 (Las1345) or *Xcc*-GFP/pBBR2 (pBBR2) cells. At day 1, a detail (right corner) of the biofilm structure in *Xcc*-GFP/Las1345 and control cells is shown. At Day 2, Biomass and Roughness coefficient using COMSTAT 2.0 were calculated. Data is shown as means ± standard deviation of three replicates. Asterisks indicate significant differences at *p* < 0.05 (Student’s *t*-test). At day 4, biofilm structure is showed by GFP (green channel) and Congo red (CR) emission (red channel) to indicate cells and exopolysaccharides production in the extracellular matrix, respectively. A detail of the biofilm structures is shown for both strains. C, GFP cells; EPS, exopolysaccharide; M, extracellular matrix. Scale bars, 20 μm.

We have previously shown that GFP-labeled *Xcc* grown in chambered cover slides, develop after 2 days microcolonies showing intimate lateral interactions, and after 4 days a typical biofilm structure (Torres et al., 2007). Under identical culture conditions, we analyzed the *in vitro* biofilm tridimensional structure of a GFP-labeled strain of *Xcc* harboring either the pBBR2::Las1345 or the pBBR2 empty vector. After 1 day of culture, *Xcc*-GFP expressing Las1345 cells were flattened over the bottom and reached the highest z-stacks positions (Las1345; 11828.21 nm vs. pBBR2; 5522.50 nm) without any observable attachment as opposed to control cells (*Xcc*-GFP/pBBR2), which were grouped at the bottom (lower z-stack) and only contacted the glass surface *via* one cell pole (Figure 6). By day two, *Xcc*-GFP/Las1345 cells shaped a homogenous layer, where few cells are interconnected, forming small cellular aggregates separated by extensive water spaces. In contrast, *Xcc*-GFP/pBRR2 cells developed more complex structures with heterogeneous sizes and shapes, in which bacteria were interconnected side-by-side (Figure 6). Biofilm structure parameters, such as biomass (volume per unit area) and roughness coefficient (biofilm heterogeneity) were analyzed by COMSTAT2. Higher biomass levels were observed in *Xcc*-GFP/Las1345 expressing Las1345, indicating an increased cell number per slide when compared with *Xcc*-GFP/pBBR2. The changing in the spatial biomass distribution can be associated with alterations in the hydrodynamic of Las1345-induced biofilm matrix that may result in enhanced availability of nutrient to the cells. In agree with this, the roughness coefficient was increased in the control (*Xcc*-GFP/pBBR2), showing an intricated biofilm matrix forming channel-like structures that may promote a hydrodynamic biofilm (Figure 6). Similar biofilm structures were also observed in the *gumB* mutant, as reported previously (Torres et al., 2007). Moreover, by day four of growth, structural changes on Las1345-expressing *Xcc* biofilms were associated with differences on the extracellular matrix disposition, surrounding cell-to-cell contacts, as shown by congo red (red) fluorescence pattern (Figure 6). Red-fluorescence signals were barely detected over the highest green network developed by *Xcc*-GFP/Las1345, suggesting that other extracellular matrix components, such as amyloid fibers or adhesins, were modified by Las1345 expression. In contrast, cultures of *Xcc*-GFP/pBBR2 cells showed extensive red areas at the cell boundaries (Figure 6). These results suggest that Las1345 expression in *Xcc* increases cell aggregates by changing extracellular matrix components to remodel biofilm structures.

We hypothesize that Las1345 could degrade intracellular precursors of the extracellular matrix, or alter the secretion of proteins or EPS to modulate biofilm development, that eventually would translate into a switch to a planktonic status, that subsequently would favor dispersal of bacteria from the biofilm structure.

**Figure 7:**
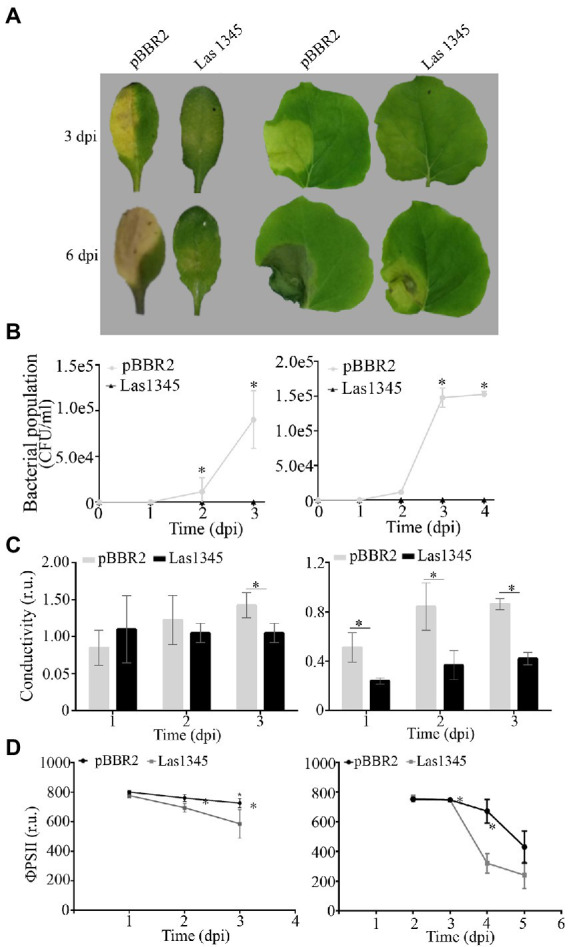
Biofilm modifications induced by Las1345 expression alters *Xanthomonas campestris* pv. *campestris* (*Xcc*) virulence. **(A)** Characterization of symptom development induced by Las1345-expresing *Xcc* in host plants. Infection of *Arabidopsis thaliana* and *Nicotiana benthamiana* with *Xcc*/Las1345 or *Xcc*/pBBR2 (10^7^ CFU/ml) using pressure infiltration. Photos of disease symptoms were taken at different days post inoculation (dpi). **(B)** Bacterial population of *Xcc* transformants in *N. benthamiana* and *A. thaliana* leaves. Values are expressed as means ± standard deviation from five samples. Each sample was obtained from three leaf disks of 1 cm^2^ from different inoculated leaves. **(C)** Quantification of cell death in leaves treated as described in (A) by measuring electrolyte leakage (conductivity) at different dpi. Values are expressed as means ± standard deviation from six samples. Each sample contains three leaf disks of 1 cm^2^ from 10 inoculated leaves. **(D)** Photosystem II quantum efficiency (ϕPSII) were measured on inoculated leaf. Values are expressed as means ± standard deviation from 15 leaves. Asterisk indicates significant differences *p* < 0.05 (Student’s *t*-test).

The presence of plant-associated biofilms is correlated with pathogenicity in *Xanthomonas*. As previously reported (Yun et al., 2006; Rigano et al., 2007; Torres et al., 2007; Conforte et al., 2019), changes in both structure and quantity of EPS reduce virulence in host plants. To further understand whether the changes in *Xcc* biofilm structure as a consequence of Las1345 expression interfere with disease progression, leaves of *A. thaliana* and *N. benthamiana* were inoculated with bacterial suspensions of *Xcc*/Las1345 and *Xcc*/pBBR2. Disease symptoms (water-soaking and necrosis) were observed in leaves inoculated with *Xcc*/pBBR2 at 3 dpi. In contrast, the disease area was reduced in leaves infected with *Xcc*/Las1345 (Figure 7A). Quantification of the bacterial population *in planta* revealed no significant differences between the *Xcc*/pBBR2 and *Xcc*/Las1345 strain growth until 36 or 48 h post inoculation (hpi), in *A. thaliana* or *N. benthamiana*, respectively (Figure 7B). However, *Xcc*/pBBR2 population gradually increased more than 10 times over the following monitoring days, while no *Xcc*/Las1345 could be recovered at soon as 3 dpi. Suppression of the *Xcc*-associated disease development was shown in *N. benthamiana* leaves infected with *Xcc gumB* mutant (Yun et al., 2006).

As compared with the areas inoculated with *Xcc*/pBBR2, *Xcc*/Las1345-infected areas had less plant cellular membrane damage, measured by electrolyte leakage, whereas photosystem II quantum efficiency (ϕPSII) was greater (Figures 7C,D), indicating that Las1345 expression compromised *Xcc*-associated disease development.

## Discussion

The identification of virulence factors that contribute to *C*Las survival both in the plant phloem as well as in the psyllid vector is necessary to understand HLB disease. In this work, we approach the function of the *C*Las serralysin-like protein Las1345 using the close relatives *R. leguminosarum* and *S. marcescens* as surrogate models. Similar to *C*Lso-serralysins (Ravindran et al., 2018), Las1345 did not show extracellular proteolytic activity in any of these two systems. Transient expression of Las1345 in *N. benthamiana* leaves did not induce phenotypic changes or alterations in the cell membrane either, which would otherwise be an indicative of protease activity. These facts, together with the absence of proteolytic activity of the protein extract from Las1345-expressing *N. benthamiana* leaves, suggest that Las1345 may not act as a protease in the plant cell.

Las1345 and its ortholog in *C*Lso were classified as serralysin-like proteins mainly based on their N-terminal zinc metalloprotease domain. However, they have only one non-classical RTX motif (GxxGND) as compared to the four well-stablished consensus motifs (GGxGxD/N) found in PrtA from *S. marcescens* or Ser from *S. liquefaciens*. Multiple RTXs are needed to maintain an unfolded or loosely-folded state in RTX proteins that enables recognition of its secretion signal by T1SS (Linhartová et al., 2010). Upon extracellular translocation, RTX proteins normally bind Ca^2+^, in a process that promotes their folding and biological activity (Linhartová et al., 2010; Zhang et al., 2012, 2015; Baumann, 2019). Studies in *Rickettsia* suggests that the requirement for secretion is the presence of tandem repeats more than the calcium binding, as RTX-ankyrin proteins which do not possess calcium binding domains can still be T1SS substrates (Jernigan and Bordenstein, 2015). These observations indicate that Las1345 could still be a substrate of T1SS, despite not containing the canonical RTX-Ca^2+^ binding motif (Linhartová et al., 2010). However, we could not detect secretion in two surrogate systems. Given the similarities between T1SSs of Gram-negative including *Ca*L spp., our current hypothesis is that Las1345 would not be secreted and would play a role in the bacterial cytoplasm. Additionally, Las1345 has an unusually large C-terminal tail (~200 aas) after the unique RTX motif that could also hinder secretion by T1SSs. In any case, secretion of Las1345 by *C*Las cannot be totally discarded as secretion assays using this bacterium cannot be performed yet. Interestingly, intracellular localization and protease activity in *R. leguminosarum* and *S. marcescens* suggested a new role for Las1345 in pathogenesis.

Intracellular localization of Las1345 raises a question regarding a possible function for this protease in the bacterial cell. The higher expression of Las1345 in *C*Las present in the citrus phloem as compared to that in the psyllid vector may be an adaptive response of this bacteria to life in every host. Las1345 might be a pathogenicity factor favoring plant colonization through the regulation of bacterial intracellular pathways. Interestingly, in bacterial surrogate models, expression of Las1345 form duller and drier microcolonies, compared with the bright and dome-shape control colonies. These changes were associated with alterations in the extracellular matrix. Biofilm is mainly composed of water (97%) and EPS including polysaccharides, lipopolysaccharides, nucleic acids, and proteins. Also, extracellular proteinaceous bacterial structures such as pili and flagella serve as structural elements that contribute to stabilize and strengthen the biofilm matrix (Limoli et al., 2015; Sena-Vélez et al., 2015). Las1345-expressing *Xcc* produces only 50% of the EPS xanthan found normally in *Xcc*. The flagellum-independent (sliding) motility is modulated by the EPS production as was shown in the xanthan deficient *gumB* mutant (Malamud et al., 2011). Here, flagella-dependent motility (swimming and swarming) is also reduced in Las1345-expressing cells. However, the *gumB* mutant showed a swimming motility similar to that of the wild type, suggesting xanthan dependent and independent modulation of biofilm for Las1345. Swarming motility is associated with the expression of flagella-associated genes in *X. citri* (Malamud et al., 2011). *C*Las genome contains most of the known flagellar genes (Duan et al., 2009; Andrade and Wang, 2019). Some of them are expressed in *C*las-infected leaves (*fliF*, *flgI*, *flgD* and *motB*) and others, particularly those involved in the hook structure (*flgK* and *flgL*) are preferentially expressed in the psyllid (Yan et al., 2013). A similar pattern was observed with genes involved in adherence. The Tad (tight adherence) pilin *flp3* gene was highly induced in psyllids and its expression is under the control of two regulators belonging to the LuxR transcriptional factor family. *C*Las LuxR regulators complement biofilm and motility deficiencies in a surrogate model (Andrade and Wang, 2019). Despite this pattern of expression, *C*Las does not produce flagella in the phloem (Yan et al., 2013; Andrade et al., 2020). The lower expression of flagellar-related genes in *Xcc*/Las1345 suggest a function for Las1345 to support the flagellum inhibition of *C*Las in the phloem, to avoid triggering of plant defense response. Moreover, the passive movement of *C*Las following the phloem sap way is the dominant bacterial movement to colonize the citrus tree (Pandey et al., 2021; Raiol-Junior et al., 2021).

Inefficient flagella assembly provokes a reduction of colony size in different mesophyll cell-infecting pathogens (Malamud et al., 2013; Berleman et al., 2016). On the other hand, flagellated phytopathogens suppress flagella expression in plants, to avoid plant defense responses (Chatnaparat et al., 2016). *Pseudomonas syringae* utilizes AprA, an extracellular metalloprotease with five RTX motifs, to degrade flagellin monomers in the extracellular milieu to avoid PTI induction (Bardoel et al., 2011; Pel et al., 2014). Plant cell death, including callose deposition and induction of defense genes, was associated with transient expression of *C*Las flagellin in *N. benthamiana* plants, suggesting that *C*Las contains in its genome factors that can trigger a PTI response (Zou et al., 2012; Shi et al., 2018). Therefore, the modulation of the expression of these factors to avoid PTI is a plausible hypothesis.

*C*Las cells were observed floating in the phloem-sap without attaching by biofilm structures (Kim et al., 2009; Hartung et al., 2010; Shokrollah et al., 2010; Hilf et al., 2013; Achor et al., 2020). In contrast, an EPS-like matrix surrounded individuals and clusters of *C*Lso cells was observed in its psyllid-vector (Cicero et al., 2016). Genome analysis indicates that *C*Las has the ability to synthesize capsular polysaccharides and surface lipopolysaccharides, suggesting that *Ca*L spp. are able to form biofilms (Cong et al., 2012; Wulff et al., 2014; Wang et al., 2017). *In vitro* biofilm formation was recently demonstrated in *L. crescens* (Lcr), the only cultured member of the genus (Fagen et al., 2014; Padgett-Pagliai et al., 2022). Lcr cell-aggregates were supported on a narrow polysaccharide matrix mainly composed by β-glucans stained with calcofluor white (Naranjo et al., 2019). The flagellum and EPS are also key elements that shape and provide structural support for bacterial biofilms in *Xanthomonas* spp. (Rigano et al., 2007; Torres et al., 2007; Malamud et al., 2011, 2013). Las1345-expressing *Xcc* established cell-to-cell contact without formation of a complex matrix which packs cells in channels-like structures. Similar biofilm modifications were observed in a xanthan-deficient mutant of *Xcc* (Torres et al., 2007). Additionally, modifications of biofilm conformation have been observed in mutants that synthesized structural variants of xanthan (Bianco et al., 2016). All these observations in a biofilm-forming bacteria like *Xanthomonas* suggest that *C*Las could somehow develop or avoid biofilm structures according to its two biological niches, as an strategy to colonize and replicate in each host. In this regard, *C*Las was grown in a biofilm and in the planktonic phase of the membrane biofilm reactor but co-cultured with another bacterial species, showing the need of *C*Las to grow in a mutualistic relationship with other bacteria according to nutrient requirements (Ha et al., 2019).

Our results suggest a new hypothesis for *C*Las pathogenesis where biofilm formation should be avoided *in planta* to favor *C*Las distribution throughout the citrus phloem. Plant intracellular pathogens may inhibit biofilm formation also as a survival mechanism to avoid the host defense response. Future studies, focused on both structure and composition of extracellular matrix using Las1345-expressing *L. crescens* should contribute to understand the lifestyle of *C*Las in its two hosts.

## Data availability statement

The original contributions presented in the study are included in the article/Supplementary material, further inquiries can be directed to the corresponding authors.

## Author contributions

Funding was acquired by MRM, JG, CG, and LG. The work was conceptualized by MRM, JG, and LG. LG, MCM, KP-P, and PT conducted the research. Data curation and formal analysis were performed by LG, MRM, and JG. RB and EGV provided mentorship and advice for research. Data curation of the published work was done by LG, MRM, and JG. All authors contributed to the article and approved the submitted version.

## Funding

This work was mainly supported by the Agencia Nacional de Promoción Científica y Tecnológica (PICT-2018-03051) to MRM and (PICT-2016-3108) to LG, by COST (European Cooperation in Science and Technology, COST Action) to JG and by the USDA National Institute of Food and Agriculture (Plant Biotic Interactions Program; award number 2017-03060) to CG.

## Conflict of interest

The authors declare that the research was conducted in the absence of any commercial or financial relationships that could be construed as a potential conflict of interest.

## Publisher’s note

All claims expressed in this article are solely those of the authors and do not necessarily represent those of their affiliated organizations, or those of the publisher, the editors and the reviewers. Any product that may be evaluated in this article, or claim that may be made by its manufacturer, is not guaranteed or endorsed by the publisher.

## Footnotes

^1^ www.comstat.dk
