# Supplementary material for: A serralysin-like protein of Candidatus Liberibacter asiaticus modulates components of the bacterial extracellular matrix
Source: Front Microbiol. 2022 Oct 19;13:1006962. doi: 10.3389/fmicb.2022.1006962 (PMC9627510; doi:10.3389/fmicb.2022.1006962)
Supplement: Supplementary file 1 [file Presentation_1.PDF]

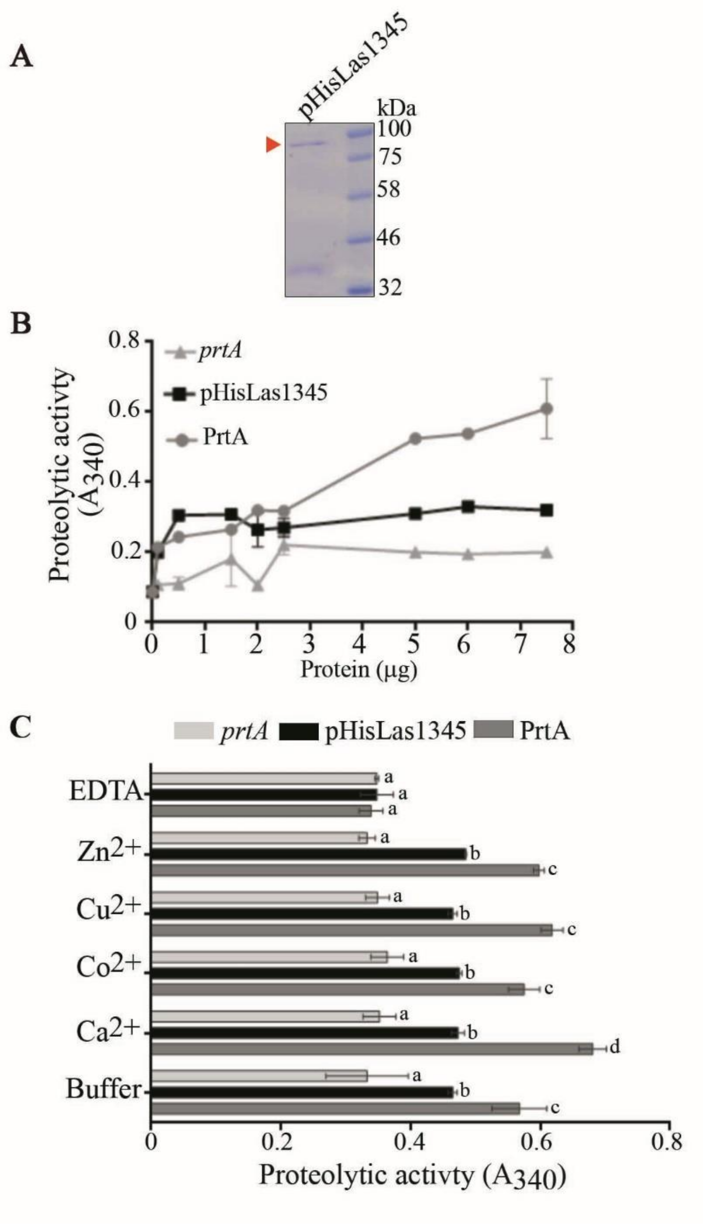

Supplementary Figure 1. Protease activity characterization of purified Las1345. (A) SDS-PAGE showing purified HisLas1345. (**B**) Protease activity of purified Las1345 (pHisLas1345) from *E. coli* compare with activity of cell free supernatant of *prtA*/pBBR2 and *prtA*/PrtA using azocasein as substrate. (**C)** Protease activity of pHisLas1345 in presence of different divalent cations or EDTA.

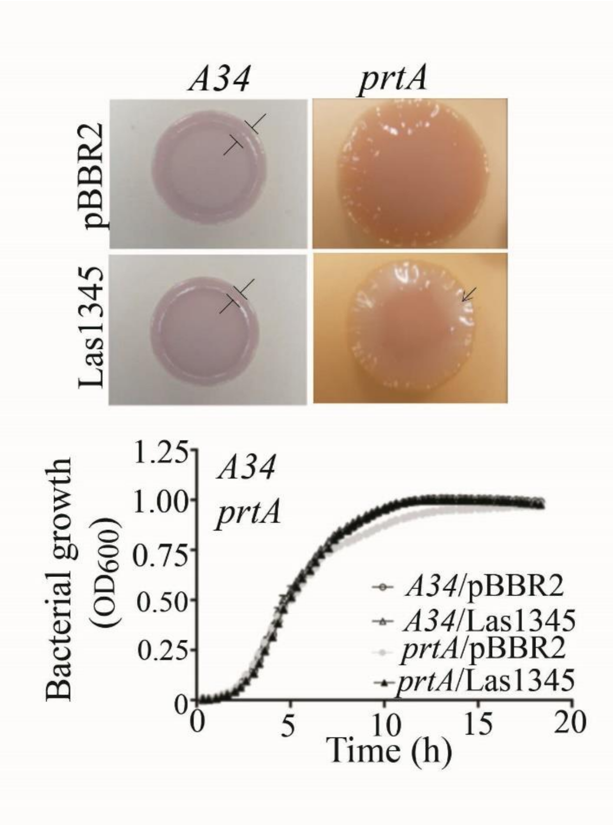

Supplementary Figure 2. Las1345 expression alters macrocolony phenotype of *Rhizobium leguminosarum* and *Serratia marcescens*. (A) Macrocolony phenotype of Las1345 expressing cells of *R. leguminosarum* A34 (*A34*) and *S. marcescens* mutant in the main protease, PrtA (*prtA*), grown for 2 days on TY and LB agar medium, respectively. Medium were supplemented with Congo Red to stain extracellular polysaccharides. Differences at boundary zone are indicated with T-markers. Changes in the polysaccharide stain is indicated by black arrow. **(B)** *A34*/Las1345 and *prtA*/Las13454 growth on TY and LB. Values are expressed as means ± standard deviation of five biological replicates. This assay was repeated five times.

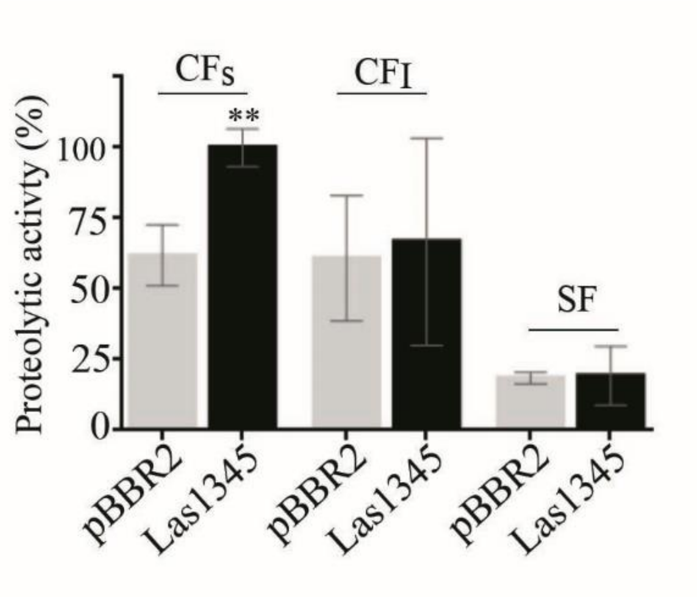

Supplementary Figure 3. Las1345 protease activity in *Xanthomonas campestris* pv. *campestris*. Protease activity in soluble and insoluble cellular fractions (CF_I and CF_S) and in the supernatant cell free fraction (SF) from *Xanthomonas campestris* pv. *campestris (Xcc)* cultures. The relative activity was expressed as the percentage of activity detected with respect to the maximum protease activity in the assay. Values are expressed as means ± standard deviations from six independent biological replicates. Asterisks indicates significant differences between *Xcc*/pBBR and *Xcc*/Las1345 at $p < 0.05$ (Student´s t-test).
